# Isolation and characterization of metaldehyde‐degrading bacteria from domestic soils

**DOI:** 10.1111/1751-7915.12719

**Published:** 2017-07-13

**Authors:** John C. Thomas, Thorunn Helgason, Chris J. Sinclair, James W. B. Moir

**Affiliations:** ^1^ Department of Biology University of York Heslington York YO10 5DD UK; ^2^ FERA Science Ltd (Fera) National Agri‐Food Innovation Campus Sand Hutton York UK

## Abstract

Metaldehyde is a common molluscicide, used to control slugs in agriculture and horticulture. It is resistant to breakdown by current water treatment processes, and its accumulation in drinking water sources leads to regular regulatory failures in drinking water quality. To address this problem, we isolated metaldehyde‐degrading microbes from domestic soils. Two distinct bacterial isolates were cultured, that were able to grow prototrophically using metaldehyde as sole carbon and energy source. One isolate belonged to the genus *Acinetobacter* (strain designation E1) and the other isolate belonged to the genus *Variovorax* (strain designation E3). *Acinetobacter* E1 was able to degrade metaldehyde to a residual concentration < 1 nM, whereas closely related *Acinetobacter* strains were completely unable to degrade metaldehyde. *Variovorax* E3 grew and degraded metaldehyde more slowly than *Acinetobacter* E1, and residual metaldehyde remained at the end of growth of the *Variovorax* E3 strain. Biological degradation of metaldehyde using these bacterial strains or approaches that allow *in situ* amplification of metaldehyde‐degrading bacteria may represent a way forward for dealing with metaldehyde contamination in soils and water.

## Introduction

**Figure 1:**
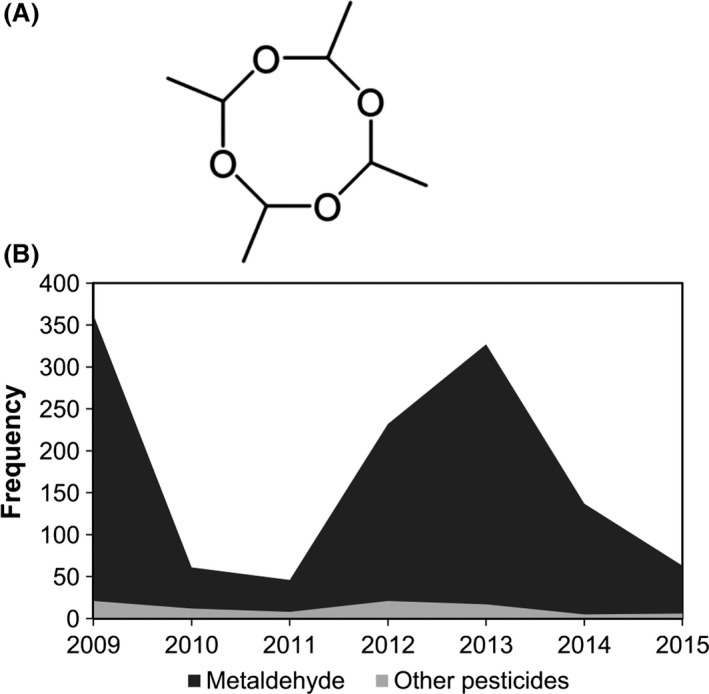
A. Skeletal structure of metaldehyde. B. Frequency of water quality failures per year in the UK due to metaldehyde or all other pesticides. Compiled from the Drinking Water Inspectorate annual regional reports, available from http://www.dwi.gov.uk/about/annual-report.

Metaldehyde (CH₃CHO)₄ is an ether, formed from a cyclic tetramerization of acetaldehyde (Fig. 1A) (Kekulé and Zincke, 1872). Metaldehyde was initially used as a solid fuel firelighter ‘Meta‐fuel’ (Miller, 1928), but its major contemporary use is as a molluscicide in agriculture and horticulture. Its application in controlling slugs was known as early as 1934 (Gimingham, 1940), and it is now widely used in both agricultural fields and domestic gardens. It is applied as a pelleted bran bait that inhibits slug feeding after exposure (Wedgwood and Bailey, 1988), causing effects such as the distention and disintegration of the Golgi apparatus and endoplasmic reticulum in the mucus cells of slugs (Triebskorn *et al*., 1998).

In 2014, Metaldehyde accounted for 87% of all recorded molluscicide applications on agricultural fields in the UK (Garthwaite *et al*., 2015). 112 tonnes were applied over 920 thousand hectares (21% of surveyed arable land used to grow crops) in Britain in 2014; primarily on wheat, oilseed rape and potato crops (Garthwaite *et al*., 2015). The vast majority of failures in drinking water quality in the UK, due to pesticide contamination, are caused by metaldehyde exceeding the regulatory limit of 0.1 μg l^−1^ (≡ 0.6 nM) (European Union Council Directive 98/83/EC) (Fig. 1B).

The recalcitrance of metaldehyde to degradation at ambient temperature (Fleischmann *et al*., 2000) is problematic for water treatment, as metaldehyde is not removed by conventional water treatment processes (Kay and Grayson, 2014). Researchers are pursuing a variety of chemical and physical approaches to deal with the problem of metaldehyde contamination (Autin *et al*., 2013; Doria *et al*., 2013; Tao and Fletcher, 2013, 2014). But currently, no economical method exists to degrade or remove metaldehyde from water.

It has been shown that the xenobiotic metaldehyde can be quickly degraded in soils (Zhang *et al*., 2011) and is oxidized to carbon dioxide under aerobic conditions in unsterilized soils (EFSA, 2010). This strongly suggests the involvement of microbes in its degradation, although no microorganisms have been isolated to date that degrade metaldehyde. The degradation of metaldehyde to CO_2_ is strongly exothermic [heat of combustion 3370 kJ mol^−1^ (Fleischmann *et al*., 2000)], suggesting that it has the potential to be a carbon and energy source to support microbial growth. Soils are home to a vast array of microbes and represent a source of metabolic activities that may be of use in industrial and medicinal applications (Delmont *et al*., 2011). Here, we enriched microbes from soils and report the first isolation and identification of microbial isolates capable of using metaldehyde as a sole source of energy and carbon for growth.

## Results and Discussion

### Two distinct metaldehyde‐degrading strains were isolated from domestic soils

Metaldehyde‐degrading bacteria were selected in a mineral medium consisting of salts Na_2_HPO_4_ (55 mM), KH_2_PO_4_ (11 mM), NH_4_Cl (6 mM) and MgSO_4_ (0.4 mM) (pH 7). This was supplemented with 2 ml l^−1^ of a trace elements solution (Vishniac and Santer, 1957). Metaldehyde was provided as sole carbon source and control cultures lacked metaldehyde. Ability to grow using metaldehyde was tested in both liquid enrichment cultures and on solid media, containing 1.5% agarose. 100 ml liquid cultures were inoculated with 1 g of soil obtained from domestic gardens in York, UK. Cultures were incubated at 30°C for 3 days, 1 ml of enrichment media was subcultured into fresh media and incubated for a further 3 days and subsequently samples were spread onto agarose plates containing 2800 μM (500 mg l^−1^) metaldehyde. Fifty to 200 colonies were obtained on plates when the enrichments were carried out in liquid culture in the presence of 570 μM (100 mg l^−1^) metaldehyde, but not following control enrichments in the absence of metaldehyde. 1 g samples of the same domestic soils were re‐suspended in 10 ml of sterile water and 100 μl aliquots spread directly onto agarose plates containing metaldehyde. Two to five colonies grew on these plates. The morphology of all the colonies was white, round and glossy. Ten isolates were picked for further analysis and named E1‐E6 and M1‐M4, to designate the source soils used. Soil E had a recent history of metaldehyde utilization, whereas soil M had not been treated with metaldehyde for at least 5 years. In each case, the isolated strains grew on agarose plates supplemented with metaldehyde, but not in its absence, suggesting they were utilizing metaldehyde as a carbon and energy source.

On subculturing the metaldehyde‐degrading strains, each strain appeared to be a pure culture, except strain E4 which yielded two distinct colony morphologies, and was subsequently subdivided into E4a and E4b. Colonies from strains E1, E3, E4a, E4b, E5, M1 and M4 were used for amplification of 16S rDNA as described previously with primers U8F and U1492R (Eden *et al*., 1991). Amplification was achieved using GoTaq polymerase (Promega) with a standard programme of: 98°C for 30 s; 35 cycles of 98°C for 10 s, 50°C for 30 s, 72°C for 60 s; 72°C for 10 min. PCR products were purified using QIAquick PCR purification kit (Qiagen, Manchester, UK) following the manufacturer's instructions. For restriction fragment length polymorphism (RFLP) analysis, 1 μg of purified DNA was digested for 1 or 3 h at 37°C using restriction enzyme HhaI. RFLP revealed two distinctly different ribotypes (see Supporting Information). Two examples of each ribotype were sequenced. Sanger sequencing was used to obtain the nucleotide sequences of the U8F‐U1492R amplicons of E1, M1, E3 and E4a using U8F as sequencing primer. Sequences from E1 and M1 were aligned using ClustalX V2.1 and found to be identical across the > 900 base region where the base sequence could be confidently assigned. Similarly, the sequences from E3 and E4a were found to be identical across a > 900 base region.

Subsequent investigation focused on the strains E1 and E3. The sequences of E1 and E3 (see Supporting Information) type strains of *A. pittii*,* A. oleivorans* and *A. seifertii* also had 99% identity to E1. The E3 sequence has 99% identity to type strains of *Variovorax boronicumulans*,* V. paradoxus*,* V. guangxiensis*,* V. ginsengisoli*. Based on these analyses, the isolates have been assigned genera and designated *Acinetobacter* E1 and *Variovorax* E3.

### The disappearance of metaldehyde from minimal media is proportional to the growth of *Acinetobacter* E1 and *Variovorax* E3 in pure cultures

**Figure 2:**
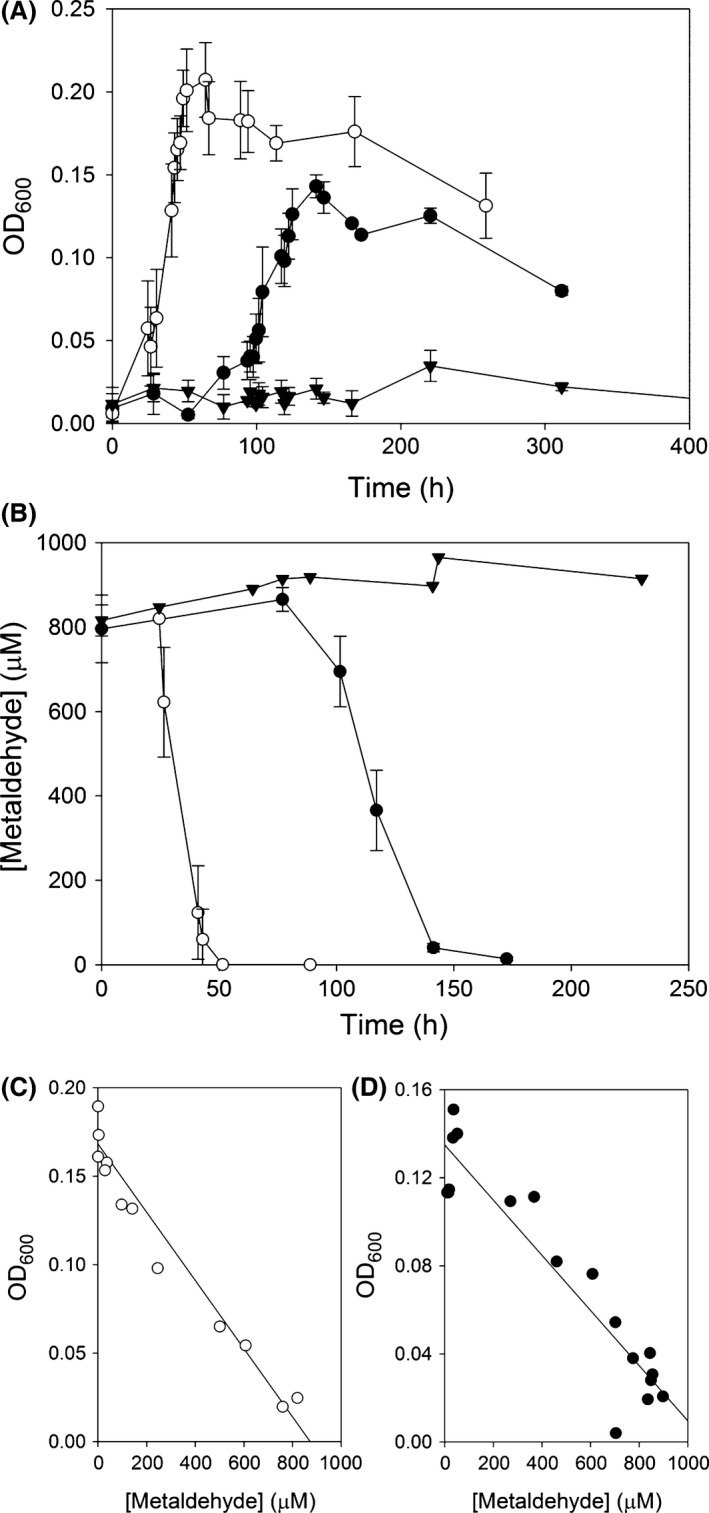
Growth and metaldehyde utilization by *Acinetobacter* E1 and *Variovorax* E3. A. Mean OD_600_ (measured using a Jenway 6300 spectrophotometer) in liquid culture with 850 μM metaldehyde as sole carbon and energy source, inoculated with single colonies of *Acinetobacter* E1 (open circles) and *Variovorax* E3 (filled circles), or not inoculated (filled triangles). Error bars give SD of triplicate independent cultures. B. Mean [metaldehyde] in culture media during growth of *Acinetobacter* E1 (open circles) and *Variovorax* E3 (filled circles), or not inoculated (filled triangles). Error bars give SD of triplicate independent cultures. Correlation between culture optical density and residual metaldehyde concentration during growth of (C) *Acinetobacter* E1 (*R* ^2^ = 0.94) and (D) *Variovorax* E3 (*R* ^2^ = 0.88) in media containing metaldehyde as the sole energy and carbon source.

Triplicate cultures of *Acinetobacter* E1 and *Variovorax* E3 were grown in minimal media with 850 μM (150 mg l^−1^) metaldehyde, incubated at 30°C with shaking at 200 rpm. An additional three flasks of media were not inoculated. Periodic samples were taken from each culture, and an uninoculated media flask and OD_600_ measurements were made. Contemporaneously, cellular material was removed from samples by centrifugation at 5000 × *g*, the supernatant aspirated and stored at −20°C for later analysis of metaldehyde content. Growth curves are shown in Fig. 2A. During the exponential growth phase, *Acinetobacter* E1 had a doubling time of 8.5 h, and *Variovorax* E3 had a doubling time of *c*. 22 h. There was no increase in optical density in the uninoculated control culture. Metaldehyde concentration of culture media samples was quantified by liquid chromatography‐mass spectrometry (for method, see Supporting Information). Metaldehyde disappeared over a similar timescale to the growth of the E1 and E3 isolates (Fig. 2B). The disappearance of metaldehyde from the cultures was correlated with the growth of the isolates (Fig. 2C and D). As the sole carbon and energy source present in the culture medium, it can be concluded that the strains were catabolizing metaldehyde for growth. *Variovorax* E3 catabolizes metaldehyde more slowly and has a longer lag time, lower maximum optical density, longer doubling time and higher final concentration of residual metaldehyde compared to *Acinetobacter* E1.

### Utilization of metaldehyde by *Acinetobacter* E1 is a property not shared by other *Acinetobacter*

The remainder of the work focused on *Acinetobacter* E1 which has faster growth kinetics, and a more rapid and complete utilization of metaldehyde, compared to *Variovorax* E3. *Acinetobacter* E1 was unable to grow using glucose, fructose, arabinose or glycerol as alternative carbon substrates.

**Figure 3:**
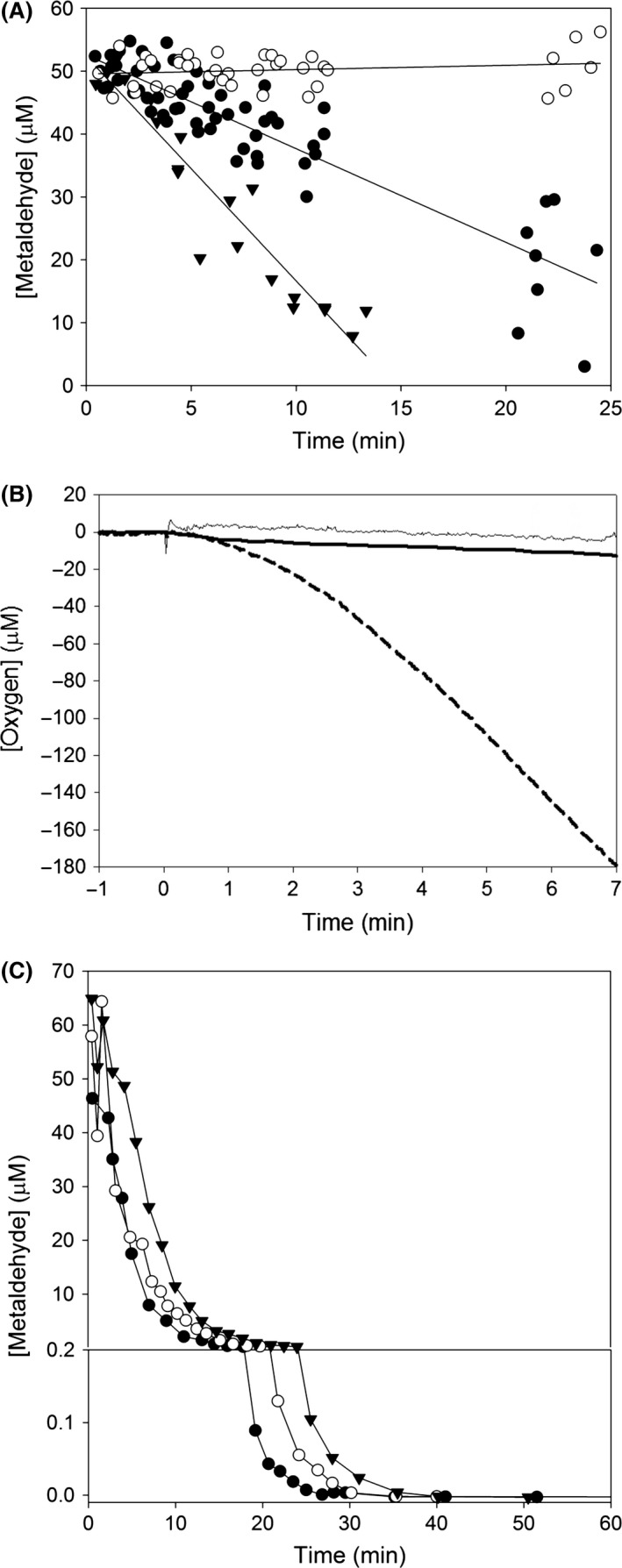
Metaldehyde utilization and metaldehyde‐dependent oxygen utilization. A. Metaldehyde utilization in samples of washed *Acinetobacter* cells resuspended to an OD_600_ = 1.0 treated with 53 μM metaldehyde following culture of *Acinetobacter* E1 in acetate (filled circles; rate of metaldehyde utilization = 1.5 ± 0.1 μM min^−1^) or in metaldehyde (filled triangles; rate of metaldehyde utilization = 3.8 ± 0.3 μM min^−1^), or strain RUH 2202 grown with acetate (open circles; rate of metaldehyde utilization = −0.1 ± 0.1 μM min^−1^) as sole carbon source. B. Metaldehyde‐dependent oxygen utilization in samples of washed *Acinetobacter* cells resuspended to an OD_600_ = 1.0 treated with 53 μM metaldehyde added at time zero. *A. calcoaceticus* RUH2202 (cultured in acetate) (solid thin line; rate of O_2_ utilization = 1.6 ± 0.4 μM min^−1^), *Acinetobacter* E1 cultured in acetate (solid thick line; rate of O_2_ utilization = 2.7 ± 1.1 μM min^−1^) or in metaldehyde (dashed line; rate of O_2_ utilization = 24.5 ± 3.8 μM min^−1^). Data are representative of at least three replicates. C. Three time‐courses of metaldehyde degradation following culture of *Acinetobacter* E1 with metaldehyde as sole carbon source. Metaldehyde axis is split to show rate of disappearance between 0–0.2 μM, and 0.2–50 μM metaldehyde.

It was desirable to identify other strains related to *Acinetobacter* E1 for comparative purposes. *A. calcoaceticus* RUH 2202 (Nemec *et al*., 2011) was purchased from the Belgian Coordinated Collection of Microorganisms, *A. calcoaceticus* ANC3678 (Nemec *et al*., 2011), *A. calcoaceticus* NIPH1 (Nemec *et al*., 1999), *A. pittii* ANC3678 (Nemec *et al*., 2011) *A. pittii* 70.29 (Seifert *et al*., 1994) and *A. baylyi* DSM14961 (Carr *et al*., 2003) from the CIP culture collection (Pasteur Institute, Paris). The ability of these *Acinetobacter* to use metaldehyde was assessed by streaking colonies from an LB plate onto a MSM + metaldehyde plate and inoculating into liquid media containing 850 μM metaldehyde. There were no signs of growth in either media after 4 days’ incubation at 30°C. *Acinetobacter* E1, unlike strain RUH 2202, was able to grow on phenol, whereas *A. calcoaceticus* RUH 2202 grew on 1% ethanol as a carbon source, but strain E1 could not grow with ethanol. Both *Acinetobacter* strains E1 and RUH 2202 grew on acetate as a carbon source, which allowed for comparative analysis of metaldehyde utilization under the same growth conditions. Following growth on acetate as sole carbon source, *Acinetobacter* E1 utilized 40 μM metaldehyde over a 30 min period, whereas there was no loss of metaldehyde in cultures of *A. calcoaceticus* RUH 2202 (Fig. 3A).

### *Acinetobacter* E1 degrades metaldehyde to completion, and this degradation is followed by oxygen consumption

Following growth on metaldehyde, *Acinetobacter* E1 utilized 40 μM metaldehyde over a 12 min period (Fig. 3A). This suggests a *c*. twofold increase in activity of the metaldehyde‐degrading enzyme following culturing with metaldehyde. Furthermore, suspensions of *Acinetobacter* E1 utilize oxygen in a metaldehyde‐dependent manner after growth on metaldehyde, but not after growth on acetate (Fig. 3B). This oxygen consumption is delayed compared to metaldehyde disappearance, indicating that the metaldehyde catabolism involves metaldehyde degradation, followed by an oxygen‐dependent metabolic step. The apparent K_M_ of cell suspensions of *Acinetobacter* E1 for metaldehyde was *c*. 50 μM, and it is noted that metaldehyde was degraded to below the limit of detection in these experiments (< 1 nM metaldehyde) in 30 min (Fig. 3C), which suggests that this or similar strains may have value in future bioremediation strategies.

Metaldehyde is a xenobiotic (i.e. only in existence due to human activity via chemical synthesis) that has been in widespread use for about 100 years. The metaldehyde‐degrading strains *Acinetobacter* E1 and *Variovorax* E3 share evolutionary heritage with other bacteria with versatile metabolism (Fewson, 1967; Willems *et al*., 1991) and a demonstrated ability to degrade xenobiotics (Mirgain *et al*., 1993; Greene *et al*., 2000; Sorensen *et al*., 2005; Wang and Gu, 2006; Bruland *et al*., 2009; Carbajal‐Rodriguez *et al*., 2011; Zhang *et al*., 2012; Rajoo *et al*., 2013; Murdoch and Hay, 2015) and other potentially recalcitrant chemicals (Reisfeld *et al*., 1972; Abbott *et al*., 1973; Koh *et al*., 1985; Hwang and Draughon, 1994; Singh and Lin, 2008; Zhao *et al*., 2009). The metabolic versatility of *Acinetobacter* and *Variovorax* isolates varies between isolates, presumably due to horizontal acquisition of genetic traits, selected in particular environments. Future work will focus on identifying the mechanistic basis for metaldehyde degradation.

To conclude, here we have demonstrated the first isolation of bacteria capable of degrading the commonly used molluscicide metaldehyde. Metaldehyde is a stable polymer of acetaldehyde which consists of a ring structure in which the bonds are aliphatic C‐C single bonds and C‐O ethers. Biological degradation of metaldehyde via the metabolic processes in bacteria such as *Acinetobacter* E1 and *Variovorax* E3 may prove valuable in dealing with metaldehyde contamination in natural environments and drinking water sources.

## Conflict of interest

None declared.

## Supporting information


**Fig. S1**. RFLP analysis of metaldehyde‐degrading bacterial isolates.
