# Supplementary material for: Isolation and characterization of metaldehyde‐degrading bacteria from domestic soils
Source: Microb Biotechnol. 2017 Jul 13;10(6):1824–9. doi: 10.1111/1751-7915.12719 (PMC5658602; doi:10.1111/1751-7915.12719)

**Supporting Information**

Measurement of metaldehyde by Liquid chromatography-mass spectrometry (LC-MS)

Liquid chromatography (LC) was performed using the Kinetex XB-C18 50 × 2.1mm column (Phenomenex, Macclesfield, UK) with 2.6 µm particles. Chromatography was performed with a flow rate of 0.5 ml/min using a mixture of 1 mM ammonium acetate prepared with ultrapure water, and methanol (J. T. Baker brand; VWR, Leicester, UK). Samples were loaded on to the column equilibrated with 10 % methanol. Subsequently, a linear gradient of [methanol] from 10 % to 90 % (v/v) was run over a three minute period. Methanol was maintained at 90 % (v/v) for a further one minute, after which time the column was re-equilibrated with 10 % methanol (v/v) for 1.5 minutes. Prepared samples were stored at 10°C prior to injection and injected onto the LC column by an autosampler. The maximum volume of filtered culture medium which could be loaded was equivalent to 0.1 μl, although typically media was diluted 50-fold with deionised water, and 5 μl loaded onto the column.

Product detection was via dual quadrupole Mass Spectrometry (MS) using the API 4000 system (SCIEX, Framingham, USA). The electrospray ionisation capillary voltage was 5250 V and desolvation gas temperature was 325 °C. During chromatography, metaldehyde is given a charge via an NH_4_^+^ adduct encountered in the mobile phase and so the precursor ion is selected at the mass of metaldehyde + ammonium: *m/z* = 194.1. Fragmentation was achieved using a collision energy of 12 eV. The product ions were observed at *m/z* = 106 and *m/z* = 62 using multiple reaction monitoring with a dwell time of 160 ms. These product ions represent fragments containing 1x acetaldehyde + ammonium and 2x acetaldehyde + ammonium.

Nucleotide sequences of 16S rRNA from *Acinetobacter* E1 and *Variovorax* E3

>Acinetobacter_E1_16S

TGCAAGTCGAGCGGAGTGATGGTGCTTGCACTATCACTTAGCGGCGGACGGGTGAGTAAT

GCTTAGGAATCTGCCTATTAGTGGGGGACAACATTTCGAAAGGAATGCTAATACCGCATA

CGTCCTACGGGAGAAAGCAGGGGATCTTCGGACCTTGCGCTAATAGATGAGCCTAAGTCG

GATTAGCTAGTTGGTGGGGTAAAGGCCTACCAAGGCGACGATCTGTAGCGGGTCTGAGAG

GATGATCCGCCACACTGGGACTGAGACACGGCCCAGACTCCTACGGGAGGCAGCAGTGGG

GAATATTGGACAATGGGCGCAAGCCTGATCCAGCCATGCCGCGTGTGTGAAGAAGGCCTT

ATGGTTGTAAAGCACTTTAAGCGAGGAGGAGGCTACTGAAGTTAATACCTTCAGATAGTG

GACGTTACTCGCAGAATAAGCACCGGCTAACTCTGTGCCAGCAGCCGCGGTAATACAGAG

GGTGCAAGCGTTAATCGGATTTACTGGGCGTAAAGCGCGCGTAGGCGGCTAATTAAGTCA

AATGTGAAATCCCCGAGCTTAACTTGGGAATTGCATTCGATACTGGTTAGCTAGAGTGTG

GGAGAGGATGGTAGAATTCCAGGTGTAGCGGTGAAATGCGTAGAGATCTGGAGGAATACC

GATGGCGAAGGCAGCCATCTGGCCTAACACTGACGCTGAGGTGCGAAAGCATGGGGAGCA

AACAGGATTAGATACCCTGGTAGTCCATGCCGTAAACGATGTCTACTAGCCGTTGGGGCC

TTTGAGGCTTTAGTGGCGCAGCTAACGCGATAAGTAGACCGCCTGGGGAGTACGGTCGCA

AGACTAAAACTCAAATGAATTGACGGGGGCCCGCACAAGCGGTGGAGCATGTGGTTTAAT

TCGATGCAACGCGAAGAACCTTACCTGGCCTTGACATAGTAAGAACTTTCCAGAGATGGA

TTGGTGCCTTCGGGAACTTACATACAGGTGCTGCATGGCTGTCGTCA

>Variovorax_E3_16S

TGCAGTCGACGGCAGCGCGGGAGCAATCCTGGCGGCGAGTGGCGAACGGGTGAGTAATAC

ATCGGAACGTGCCCAATCGTGGGGGATAACGCAGCGAAAGCTGTGCTAATACCGCATACG

ATCTACGGATGAAAGCAGGGGATCGCAAGACCTTGCGCGAATGGAGCGGCCGATGGCAGA

TTAGGTAGTTGGTGAGGTAAAGGCTCACCAAGCCTTCGATCTGTAGCTGGTCTGAGAGGA

CGACCAGCCACACTGGGACTGAGACACGGCCCAGACTCCTACGGGAGGCAGCAGTGGGGA

ATTTTGGACAATGGGCGAAAGCCTGATCCAGCCATGCCGCGTGCAGGATGAAGGCCTTCG

GGTTGTAAACTGCTTTTGTACGGAACGAAACGGCCTTTTCTAATAAAGAGGGCTAATGAC

GGTACCGTAAGAATAAGCACCGGCTAACTACGTGCCAGCAGCCGCGGTAATACGTAGGGT

GCAAGCGTTAATCGGAATTACTGGGCGTAAAGCGTGCGCAGGCGGTTATGTAAGACAGTT

GTGAAATCCCCGGGCTCAACCTGGGAACTGCATCTGTGACTGCATAGCTAGAGTACGGTA

GAGGGGGATGGAATTCCGCGTGTAGCAGTGAAATGCGTAGATATGCGGAGGAACACCGAT

GGCGAAGGCAATCCCCTGGACCTGTACTGACGCTCATGCACGAAAGCGTGGGGAGCAAAC

AGGATTAGATACCCTGGTAGTCCACGCCCTAAACGATGTCAACTGGTTGTTGGGTCTTCA

CTGACTCAGTAACGAAGCTAACGCGTGAAGTTGACCGCCTGGGGAGTACGGCCGCAAGGT

TGAAACTCAAAGGAATTGACGGGGACCCGCACAAGCGGTGGATGATGTGGTTTAATTCGA

TGCAACGCGAAAAACCTTACCCACCTTTGACATGTACGGAATTCGCCAGAGATGGCTT

Figure 2. RFLP analysis of metaldehyde-degrading bacterial isolates. 16S rRNA amplicons of different isolates were digested by HhaI and separated by agarose gel electrophoresis. Lane 1, NEB 100 bp ladder; 2, E1; 3, E3; 4, E4a; 5, E4b; 6, M1; 7, E5; 8, M4.


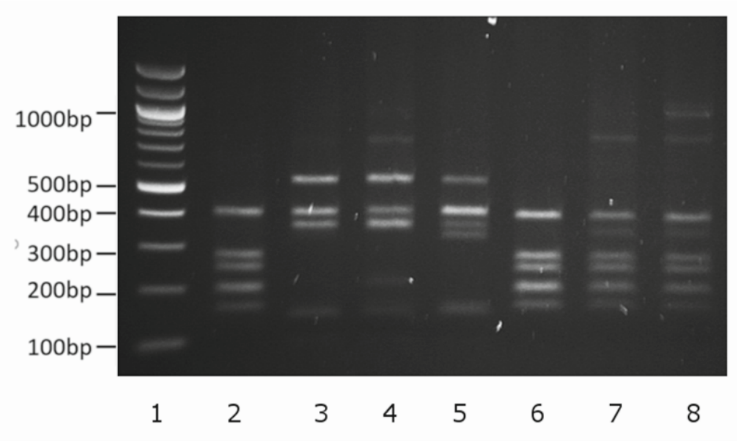

Supplement: Supplementary file 1 — Fig. S1. RFLP analysis of metaldehyde‐degrading bacterial isolates. [file MBT2-10-1824-s001.docx]
